# Occurrence of Cryptosporidium Infection and Associated Risk Factors among HIV-Infected Patients Attending ART Clinics in the Central Region of Ghana

**DOI:** 10.3390/tropicalmed6040210

**Published:** 2021-12-09

**Authors:** Kwabena Dankwa, Samuel Victor Nuvor, Dorcas Obiri-Yeboah, Patrick Kwame Feglo, Mohamed Mutocheluh

**Affiliations:** 1 Department of Clinical Microbiology, Kwame Nkrumah University of Science and Technology, Private Mail Bag, University Post Office, Kumasi, Ghana; pfeglo@gmail.com (P.K.F.); mutocheluh@gmail.com (M.M.); 2 Department of Microbiology and Immunology, University of Cape Coast, Private Mail Bag, University Post Office, Cape Coast, Ghana; s.v.nuvor@uccsms.edu.gh (S.V.N.); d.obiri-yeboah@uccsms.edu.gh (D.O.-Y.); 3 Directorate of Research, Innovation, and Consultancy, University of Cape Coast, Private Mail Bag, University Post Office, Cape Coast, Ghana

**Keywords:** *Cryptosporidium*, risk factors, HIV, Ghana, Central region

## Abstract

*Cryptosporidium* species are intestinal protozoan parasites that infect and cause diarrhoea in animals and humans. The current study was conducted to determine the prevalence and risk factors of *Cryptosporidium* infection among HIV-infected patients in the Central region of Ghana. In this cross-sectional study, four hundred eighteen documented HIV-infected participants from four health facilities that provide antiretroviral therapy (ART) services across the Central region of Ghana were selected by systematic random sampling. An enzyme-linked immunosorbent assay (CoproELISA^TM^, *Cryptosporidium* Savyon^®^ Diagnostics Ltd., Ashdod, Israel) was used to detect *Cryptosporidium* antigens in stool samples obtained from participants. Information regarding participants’ sociodemographic characteristics and clinical symptoms as well as potential environmental and behavioral risk factors were collected using a structured questionnaire. Chi-square or Fisher’s exact tests were used to determine associations between *Cryptosporidium* infections and explanatory variables, while risk factors were assessed using multivariate logistic regression analysis. The overall prevalence of *Cryptosporidium* infection among HIV-infected participants in this study was 6.2% (95% CI: 3.90–8.54). *Cryptosporidium* was not significantly associated with any of the sociodemographic variables, patient clinical symptoms, and environmental factors. However, the prevalence of the parasite was significantly higher 25% (95% CI: 1.17–48.83; *p* = 0.013) among participants who did not always wash their hands before meals and those who did not always wash vegetables before eating them, 23.5% (95% CI: 1.05–46.01; *p* = 0.016). Multivariate logistic regression analysis showed that participants who used public water closet facilities were approximately 9 times more likely to become infected with the parasite than those who practised open defecation (OR: 8.83; 95% CI: 1.22–64.13; *p* = 0.031). In conclusion, *Cryptosporidium* is prevalent among HIV-infected patients in the Central region of Ghana. An important risk factor identified was the use of the public water closet toilet facility. More attention should be given to ensuring cleanliness at shared water closet facilities in addition to adequate disinfection of hands after using such facilities.

## 1. Introduction

*Cryptosporidium* species are apicomplexan parasites that infect man and other vertebrates [1,2]. Currently, more than 38 species of the parasite have been described worldwide with *Cryptosporidium hominis* and *Cryptosporidium parvum* implicated in most human infections [3,4]. *Cryptosporidium* infection in immunocompetent hosts is usually self-limiting but can be severe and prolonged in immunocompromised individuals [5]. 

Infections caused by *Cryptosporidium* species partly contribute to the high number of diarrhoea cases among immune-compromised persons and children [6,7,8]. The Global Enteric Multicenter Study (GEMS) revealed the parasite as a significant contributor to the diarrhoea burden in children less than two years old in sub-Saharan Africa [9]. This is because of the ubiquitous and robust nature of the oocysts of the parasite, which is transmitted either directly from person to person or indirectly through vehicles such as contaminated food or water, or from animals to humans [10,11].

*Cryptosporidium* has been reported in several studies among HIV-positive individuals around the world with a prevalence ranging from 2.17% to 55% in China, Uganda, Kenya, Congo Democratic Republic, Ethiopia, and Nigeria [12,13,14,15,16,17,18]. In Ghana, hospital-based studies among HIV-infected participants in the Ashanti and Central regions have reported prevalences of 14% and 46%, respectively [19,20].

Methods available for the detection of *Cryptosporidium* infections in stool samples include conventional microscopy to identify oocysts on stained slides and immunological assays based on the detection of *Cryptosporidium* antigens by immunochromatography or enzyme-linked immunosorbent assay (ELISA). Currently, molecular methods such as real-time polymerase chain reaction (RT-PCR), polymerase chain reaction-restriction fragment length polymorphism (PCR-RFLP), and PCR—sequencing protocols are used in the identification of genotypes/species of the parasite [21,22,23]. It must however be noted that most of the studies on *Cryptosporidium* in Ghana utilized conventional microscopy [6,18,20]. Although ELISA has proven in many cases to be more sensitive in the detection of *Cryptosporidium* species than microscopy, its use is limited to a few studies in Ghana [24,25,26].

Globally, HIV infections currently stand at about 37.6 million people with adults (15 years and above) constituting 95% of the infected population [27]. In Ghana, the adult HIV prevalence is estimated at 1.7%, with less than half of them receiving anti-retroviral drugs, a situation which makes the HIV-infected population vulnerable to many infections, including *Cryptosporidium* [27,28,29]. Some of the risk factors that contribute to *Cryptosporidium* infection, in general, are related to contaminated food and drinking water sources, contact with infected animals, crowded living conditions and contact with children in diapers [20,30,31]. However, potential risk factors for *Cryptosporidium* infection among HIV-infected persons in the Central region are poorly understood. The only study that explored the problem was limited with sample size but implicated the source of drinking water as a risk factor [20]. The goal of this study was to determine the prevalence and risk factors of *Cryptosporidium* infection among HIV-infected patients in the Central region of Ghana.

## 2. Materials and Methods

### 2.1. Study Design/Sites

**Figure 1:**
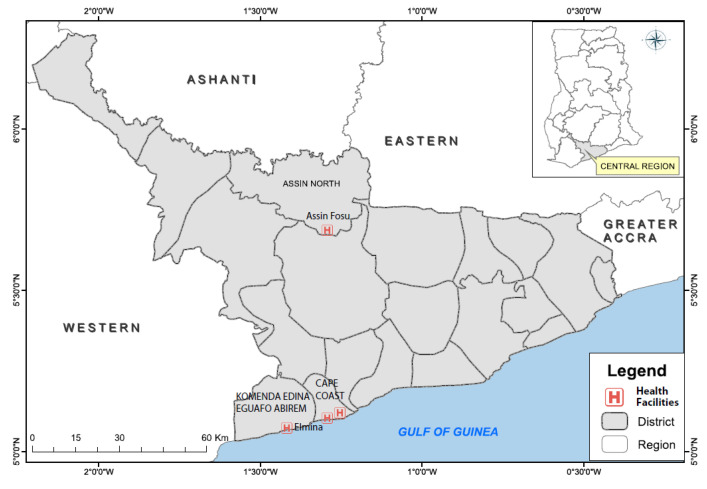
Map of the Central region of Ghana, showing the location of study facilities.

This was a health facility-based cross-sectional study carried out in the Central region, one of Ghana’s 16 political administrative areas occupying a total land area of 9826 square kilometers with a population of approximately 2,201,863 people [32]. The region lies across the coastal and forest ecological zones in the southern part of Ghana with a local economy based on fishing, farming, tourism, and trading, among others. The four health facilities included in this study were the HIV clinics of The Cape Coast Teaching Hospital, St Francis Xavier Hospital at Assin Foso, Ewim Polyclinic in Cape Coast, and the Elmina Polyclinic at Elmina (Figure 1). These facilities offer medical care and antiretroviral therapy (ART) services to many registered HIV patients in the region and were purposely selected because of the large number of patients they serve. The clinic at the Cape Coast Teaching hospital is a referral facility, which provides specialized services to registered HIV patients across the region and beyond. 

### 2.2. Study Population/Sampling/Sample Size

The study involved documented HIV-infected patients with or without diarrhoea attending HIV clinics in the selected health facilities. Participants were recruited by systematic random sampling. A sample interval was determined based on the total number of patients at the clinic. A participant was then chosen among the first three at random. Every other third participant was then selected for the study. The eligibility criteria for participants included being 18 years of age or older, being resident in the Central region, having the ability to provide a stool sample and answer a questionnaire. As reliable data on *Cryptosporidium* infections among HIV/AIDS patients 18 years or older were not readily available, the prevalence was estimated at 50% with a margin of error of 5%, at 95% level of confidence, and an expected response rate of 0.9 by Cochran’s formula [33]. A minimum number of 384 participants was required, but in all 418 participants took part in the study. The number of participants allocated per study facility was based on the number of patients seen in the clinic within 3 months before the study. 

### 2.3. Sample Collection

A single fresh stool sample was obtained from each participant at the various health facilities included in the study between December 2018 and July 2019. The participants were given labelled screw-capped plastic containers with a spoon for the collection of the samples. They were also given instructions on how to collect the samples without it getting contaminated with urine. The samples were kept on ice and sent to the laboratory of the School of Medical Sciences, University of Cape Coast where a portion of each was preserved in 10% formalin for further analysis as was required by the commercial ELISA kit used in the study. A portion of the stool sample was also stored at −20 °C for future molecular work. 

### 2.4. Questionnaire Administration

A structured questionnaire was administered to the study participants to identify risk factors associated with *Cryptosporidium* infection. The questionnaire captured socio-demographic information, the clinical symptoms within 2 weeks before sample collection as well as some environmental and behavioural risk factors of participants. Information on whether participants were on antiretroviral therapy and duration was also obtained.

### 2.5. Detection of Cryptosporidium Stool Antigens

The study utilized a commercial enzyme-linked immunosorbent assay stool kit (CoproELISA^TM^ *Cryptosporidium,* Savyon^®^ Diagnostics Ltd., Ashdod, Israel) for the qualitative detection of *Cryptosporidium* antigens in participants stool samples. The procedure outlined by the manufacturer was duly followed using the 10% formalin preserved stool samples. Briefly, all components of reagents used in the test were brought to room temperature. For each test, one positive and two negative controls were always run along with the samples in a 96 well microtiter plate. A dilution tube was set up for each formalin preserved stool sample in a 1.5 mL Eppendorf tube where 0.15 g or 0.15 mL of stool sample was added to 400 μL of stool diluent using a disposable collector. The faecal specimen was then vortexed and left to stand for at least 10 min but not more than 30 min to allow large particles of materials to precipitate.

One hundred microliters each of the positive control, negative controls in duplicate, and the same volume of diluted samples were dispensed into the wells of the microtiter plate (coated with anti-cryptosporidium antibodies) using disposable pipettes. The plate was then incubated at 37 °C in a moisture chamber for 1 h. This was followed by a washing step with 300 μL of wash buffer 5 times after which 100 μL of the conjugate was then added to each well and incubated for 1 h at 37 °C in a moisture chamber. The ELISA plates were then washed with buffer 5 times.

Finally, 100 μL of TMB-substrate was added to each well and incubated at room temperature for 15 min after which the reaction was stopped by adding 100 μL of stop solution (1M H_2_SO_4_) into each well. The absorbance at 450 nm was measured with a Multiskan EX ELISA plate reader(Thermo Scientific, Waltham, MA, USA. The results were then printed out and used in interpreting the test. A cut-off value was determined using the mean absorbance value of the negative control plus 0.3. The results of the test were interpreted as negative (*Cryptosporidium* antigens not detectable) when the optical density of the sample was less than the cut-off value and positive (detectable *Cryptosporidium* antigens) when it was equal to or more than the cut-off value. 

### 2.6. Ethical Considerations

The study protocol was reviewed and approved by the Committee on Human Research Publication and Ethics at the Kwame Nkrumah University of Science and Technology in Kumasi, Ghana (CHRPE/AP/550/18), and the Ethical Review Committee of the Cape Coast Teaching Hospital (CCTHERC/EC/2018/4) before commencement. Permission was also obtained from the other participating health facilities. The goals of the study were explained to all participants who were included only after they gave written informed consent. In addition, the administration of the questionnaire was done in designated offices of the health facilities to ensure privacy, guarantee confidentiality and avoid any distractions to participants. 

### 2.7. Statistical Analysis

Data from the questionnaire and corresponding results of the laboratory tests of the participants were entered into IBM’s SPSS statistics for windows version 25.0 (IBM Corp.: Armonk, NY, USA) for statistical analysis. Continuous variables were summarized using descriptive statistics such as the mean and standard deviation while for categorical variables we used frequencies and proportions. The associations between *Cryptosporidium* infection and sociodemographic and other explanatory variables were assessed using Pearson’s Chi-square or Fisher’s exact test where appropriate. The risk factors were assessed using multivariate logistic regression analysis. Odds ratios were determined at 95% confidence intervals (CI) in the model to measure the strength of association between the dependent and independent variables. In these analyses, *p* ≤ 0.05 was considered statistically significant.

## 3. Results

### 3.1. Sociodemographic Characteristics of Participants 

A total of 418 HIV-positive patients were involved in the study. The mean age of the participants was 45.8 (±11.7) years while the range was between 18 and 77 years. A greater proportion of participants in the study (41.1%) were in the 45–59-year age bracket. There were more females (81.6%) than males, while most participants were involved in trading (39.2%). Approximately a quarter of participants (25.4%) in the study had no formal education while the majority (60.8%) had household size with 5 members or less. Additionally, most participants (70.8%) were residents in urban areas while only a few (5.5%) had yet to receive antiretroviral treatment (Table 1).

### 3.2. Prevalence and Association between Cryptosporidium Infection and Participants Characteristics and Symptoms 

The overall prevalence of *Cryptosporidium* infection in this study was 6.2% (95% CI: 3.90–8.54). Generally, there was no significant association between *Cryptosporidium* infection and any of the sociodemographic variables (Table 2). The prevalence of *Cryptosporidium* was higher in males (10.4%), among those who were not working (8.7%), those in the 30–44-year category (9.4%) and participants with no formal education (8.5%). However, the prevalence rate of the parasite was lower (5.5%) among participants with more than 5 members in their households and those residing in rural areas (5.7%). 

With regards to the clinical symptoms of participants and infection with the parasite, there was no association. The prevalence of *Cryptosporidium* infection was however higher among participants with nausea (7.1%) and abdominal pains (7.5%). The prevalence was also higher (8.4%) among participants who had been on antiretroviral treatment between 1–12 months (Table 3).

### 3.3. Association between Environmental, Behavioral Risk Factors and Cryptosporidium Infection

There were no associations between any of the environmental factors and *Cryptosporidium* infection (Table 4). Participants who used tap water as main source for drinking, those who used public water closets and those who had pets at home had higher rates of infection. *Cryptosporidium* infection was associated with some behavioural factors of participants in the study such as not washing hands before meals (*p* = 0.013) and not washing vegetables before eating them (*p* = 0.016) (Table 5). There was however no association between washing fruits before eating, washing hands with soap after visiting the toilet and changing children’s diapers.

### 3.4. Risk Factors for Cryptosporidium Infection 

In the multivariate logistic regression analysis (Table 6), the use of public water closet toilet facilities by participants was the only significant predictor for *Cryptosporidium* infection. Participants who used public water closets had higher odds of becoming infected with *Cryptosporidium* than those who practiced open defecation (OR 8.83; CI: 1.22–64.13, *p* = 0.031) Participants who did not always wash their hands before meals, those who did not always wash vegetables before eating and the others who indicated that did not always wash fruits before eating them also had increased risks of *Cryptosporidium* infection, but these were not statistically significant.

## 4. Discussion

*Cryptosporidium* species are a common cause of diarrhoea among HIV patients, other immunocompromised individuals as well as in immunocompetent persons. Information about the parasite and factors associated with its infection, especially among HIV-infected individuals in Ghana is however limited. This study was carried out to determine the occurrence and associated risk factors of *Cryptosporidium* species among HIV-infected patients in the Central region of Ghana using an antigen detecting enzyme-linked immunosorbent assay.

The overall prevalence of *Cryptosporidium* infection among the HIV-infected population in this study was 6.2%. Studies carried out earlier in different parts of the country, showed varied levels of prevalence of the parasite. The occurrence of the parasite in the current study is lower than that in studies conducted in other parts of Ghana such as Cape Coast, Kumasi, Accra, and Tarkwa, which reported prevalence of between 11% and 46% [6,19,20,34,35]. The prevalence of *Cryptosporidium* infection in the current study is however higher than that in a similar study on intestinal parasites and HIV/AIDS coinfections in Offinso and Nyinahin hospitals in the Ashanti region of Ghana which recorded a *Cryptosporidium* prevalence of 2.1% [18]. Other studies among HIV individuals in Mozambique, Kenya, Thailand, Iran, and Ethiopia have reported prevalence between 8.9% and 26.3% [14,16,36,37,38], which were higher than those obtained in the present study. However, the prevalence in the current study was higher compared to studies in Nigeria, North India, and the Democratic Republic of Congo [39,40,41]. Several explanations may account for the varying prevalence recorded in various studies of the parasite. These include factors such as whether the study population had diarrhoea or not or whether participants were on highly active antiretroviral therapy, the diagnostic method used, the season of sampling and location of study, among others. It is also likely that the prevalence of *Cryptosporidium* infection may not depend on all these factors but some of them. 

The relatively low prevalence of the parasite in the present study could also be because of the high proportion of participants on highly active antiretroviral therapy (HAART). Similar studies from Ghana and other countries among HIV-positive patients on HAART treatment have reported significant improvement in their immunological status [42,43]. Thus, therapeutic measures aimed at improving the immune status of HIV-infected patients contribute significantly to reducing the prevalence of the parasite.

The detection method of the parasite could also have accounted for the level of prevalence recorded in the present study. A previous study in the Central region of Ghana using the modified Ziehl Neelsen (ZN) staining method reported a *Cryptosporidium* prevalence of 46% which was quite high, but one of the strengths of the current study is the large sample size [20]. It must be pointed out that even though the modified Ziehl Neelsen (ZN) method is most widely used for detecting *Cryptosporidium* in Ghana and other places in Africa [44], the sensitivity of the ELISA technique is reported to be superior with the capacity to detect *Cryptosporidium* antigens even in the absence of oocysts [25]. 

In addition, a higher prevalence of *Cryptosporidium* is usually associated with individuals presenting with diarrhoea. However, the prevalence of the parasite in the current study was lower than that in an earlier study in a tertiary hospital in Ghana which involved HIV-infected patients with chronic diarrhoea and reported a 46% prevalence rate [20]. This may be because only approximately a quarter of participants in the current study reported having diarrhoea.

The lack of association between *Cryptosporidium* infection and the various factors in our study may be because of improvement in the immune status of the patients. This is because most of the participants in the study were on antiretroviral therapy, which reduces the impact of the disease on affected individuals as it improves their CD4+ T-lymphocyte levels and reduces their viral load [45]. However, *Cryptosporidium* infection was found to be significantly higher among participants who did not wash their hands before meals as well as those who did not always wash vegetables before eating them. Contaminated hands resulting from inadequate hand hygiene facilitate the transmission of the parasite as was reported in a study among veterinary students following the handling of animals on the farm [46]. Hand washing before meals is therefore important in preventing contamination of food and ingestion of oocysts of the parasite. 

The observation of an association between *Cryptosporidium* infection and participants not washing vegetables is similar to the findings of a study in Spain [47]. There are some studies in Ghana and other countries that also reported the contamination of vegetables with oocysts of the parasite [47,48,49]. The oocysts are hardy and readily infectious immediately they are passed out by the host into the environment and only adequate disinfection of the vegetables can prevent consumers from getting infected.

Participants who used public water closets were approximately 9 times more likely to be infected with *Cryptosporidium* than those who practiced open defecation. This finding is quite surprising as the use of water closet is apparently more hygienic than the practice of open defecation. Even though the water closet is considered as an ‘improved sanitation’ facility, a general lack of cleanliness that characterizes most of the shared public toilet facilities [50] may account for this observation. Shared toilets have been reported to be associated with diarrhoea and other diseases in Uganda [51] and are therefore classified as limited sanitation by the WHO/UNICEF-Joint Monitoring Program (JMP) for water supply and sanitation because of their unhygienic and poorly managed nature [52].

It must also be noted that this study was not without any limitations. Key among them was the detection of *Cryptosporidium* antigens using ELISA which did not offer any information on the species of the parasite involved. We were, therefore, unable to ascertain whether some of the infecting species were zoonotic. Employing molecular detection methods in future studies would help in the identification of species/genotypes and sub-types of the parasite. Again, no information was collected on the nature and state of hand hygiene facilities available at these public toilet facilities, and this should also be addressed in future studies.

## 5. Conclusions

*Cryptosporidium* species is prevalent among HIV-infected patients in the Central region of Ghana at 6.2%. The use of the public water closet facility was identified as an important risk factor. We recommend that more attention be given to shared public water closet toilet facilities in terms of cleanliness. HIV-infected patients should also ensure adequate disinfection of hands after visiting such facilities. 

## Figures and Tables

**Table 1:** Socio-Demographic Characteristics of Study Participants.

Characteristic	Mean (SD)/Range/n (%)
Age (years)	
Mean	45.8 (±11.7)
Range	18–77
≤29	34 (8.1)
30–44	149 (35.7)
45–59	172 (41.1)
>59	63 (15.1)
Sex	
Male	77 (18.4)
Female	341 (81.6)
Occupation	
Not working	46 (11.0)
Farming/Fishing	119 (28.5)
Trading	164 (39.2)
Others	89 (21.3)
Educational level	
No formal education	106 (25.4)
Primary	91 (21.8)
Middle/Junior High	158 (37.7)
Senior High and above	63 (15.1)
Size of Household	
≤5 members	254 (60.8)
>5 members	164 (39.2)
Location	
Rural	122 (29.2)
Urban	296 (70.8)
Duration on ART	
0 month *	23 (5.5)
1–12 months	119 (28.5)
≥12 months	276 (66.0)

* Participants were not yet on HAART.

**Table 2:** Prevalence of *Cryptosporidium* infection and Association with Sociodemographic Characteristics of Participants.

Variables	Number of Respondents	Positives (%)	95% CI	*p*-Value
ELISA results	418	26 (6.2)	3.90–8.54	-
Sex				
Male	77	8 (10.4)	3.42–17.36	0.114
Female	341	18 (5.3)	2.89–7.66	
Age in years				
≤29	34	1 (2.9)	−3.04–8.93	0.255
30–44	149	14 (9.4)	4.66–14.14	
45–59	172	8 (4.7)	1.47–7.83	
>59	63	3 (4.8)	−0.64–10.17	
Occupation				
Not working	46	4 (8.7)	0.24–17.16	0.795
Farming and fishing	119	7 (5.9)	1.59–10.17	
Trading	164	11 (6.7)	2.84–10.58	
Others	89	4 (4.5)	0.11–8.88	
Educational level				
No formal education	106	9 (8.5)	3.10–13.88	0.577
Primary	91	5 (5.5)	0.72–10.27	
Middle/Junior High	158	10 (6.3)	2.49–10.17	
Senior High/Tertiary	63	2 (3.2)	−1.28–7.63	
Size of Household				
≤5 members	254	17 (6.7)	3.54–9.64	0.683
>5 members	164	9 (5.5)	1.93–8.85	
Location				
Rural	122	7 (5.7)	1.59–9.92	0.793
Urban	296	19 (6.4)	3.61–9.23	

**Table 3:** Association between Clinical Symptoms, Duration on ART and *Cryptosporidium* Infection.

Variables	Number of Respondents (%)	Positives (%)	95% CI	*p*-Value
Diarrhoea				
YES	102 (24.4)	6 (5.9)	1.24–10.53	0.871
NO	316 (75.6)	20 (6.3)	3.63–9.03	
Nausea				
YES	84 (20.1)	6 (7.1)	1.52–12.77	0.695
NO	334 (79.9)	20 (6.0)	3.43–8.55	
Vomiting				
YES	39 (9.3)	2 (5.1)	−2.12–12.37	0.761
NO	379 (90.7)	24 (6.3)	3.87–8.80	
Abdominal pains				
YES	120 (28.7)	9 (7.5)	2.72–12.28	0.492
NO	298 (71.3)	17 (5.7)	3.06–8.35	
Duration on ART				
0 month	23 (5.5)	1 (4.3)	−4.67–13.36	0.496
1–12 months	119 (28.5)	10 (8.4)	3.35–13.46	
>12 months	276 (66.0)	15 (5.4)	2.74–8.13	

**Table 4:** Association between Environmental Factors and *Cryptosporidium* Infection.

Variables	Number of Respondents (%)	Positives (%)	95% CI	*p*-Value
Drinking water source				
Tap	227 (54.3)	16 (7.1)	3.69–10.4	0.936
Sachet	135 (32.3)	7 (5.2)	1.40–8.97	
Borehole	15 (3.6)	1 (6.7)	−7.63–20.97	
Well	26 (6.2)	1 (3.9)	−4.08–11.77	
Stream	15 (3.6)	1 (6.7)	−7.63–20.97	
Toilet facility used				
Water closet (private)	81 (19.4)	7 (8.6)	2.34–14.89	0.132
Water closet (public)	18 (4.3)	3 (16.7)	−2.40–35.74	
KVIP/Pit latrine	252 (60.3)	14 (5.6)	2.71–8.40	
Open defecation	67 (16.0)	2(2.99)	−1.20–7.17	
Livestock at home				
YES	287 (68.7)	16 (5.6)	2.90–8.25	0.419
NO	131 (31.3)	10	3.03–12.24	
Pets at home				
YES	306 (73.2)	20	3.75–9.32	0.659
NO	112 (26.8)	6	1.12–9.59	

KVIP—Kumasi Ventilated Improved Pit latrine.

**Table 5:** Association between Behavioral Factors and *Cryptosporidium* Infections.

Variable	Number of Respondents (%)	Positives (%)	95% CI	*p*-Value
Wash hands always before meals				
YES	402 (96.2)	22 (5.5)	3.24–7.71	* 0.013
NO	16 (3.8)	4 (25.0)	1.17–48.83	
Wash fruits always before eating				
YES	246 (58.9)	11 (4.5)	1.87–7.07	0.077
NO	172 (41.1)	15 (8.5)	4.46–12.98	
Wash vegetables always before eating them				
YES	401 (95.9)	22 (5.5)	3.25–7.72	* 0.016
NO	17 (4.1)	4 (23.5)	1.05–46.01	
Wash hands with soap after visiting the toilet				
YES	401 (95.9)	25 (6.2)	3.86–8.61	1.000
NO	17 (4.0)	1 (5.9)	−6.59–18.4	
Change diapers of young children				
YES	109 (26.1)	7 (6.4)	1.75–11.10	0.919
NO	309 (73.9)	19 (6.2)	3.46–8.84	

* Statistically significant at *p* ≤ 0.05.

**Table 6:** Multivariate analysis of risk factors for *Cryptosporidium* Infection.

Variables	Prevalence (%)	Odds Ratio (95% CI)	*p-*Value
Sex			
Male	10.4	2.08(0.83–5.23)	0.118
Female	5.3	1	
Toilet facility used regularly			
Water closet (private)	8.6	3.23(0.58–15.59)	0.171
Water closet (public)	16.7	8.83(1.22–64.13)	0.031 *
KVIP/Pit latrine	5.6	2.20(0.46–10.45)	0.320
Open defecation	3.0	1	
Always wash hands before meals			
No	25.0	2.43(0.54–10.97)	0.248
Yes	5.5	1	
Always wash vegetables before eating			
No	23.5	3.21(0.71–14.64)	0.132
Yes	5.5	1	
Always wash fruits before eating			
No	8.7	1.77 (0.75–4.19)	0.195
Yes	4.5	1	

* Statistically significant at *p* ≤ 0.05, KVIP—Kumasi ventilated improved pit latrine.

## Data Availability

The data presented in this study are available on request from the corresponding author.
